# Is Xpert MTB/RIF appropriate for diagnosing tuberculous pleurisy with pleural fluid samples? A systematic review

**DOI:** 10.1186/s12879-018-3196-4

**Published:** 2018-06-25

**Authors:** Zhen-yu Huo, Li Peng

**Affiliations:** Department of Respiratory and Critical Care Medicine, the First Affiliated Hospital of Chongqing Medical University, No.1, Youyi Road, Yuzhong District, Chongqing Municipality, 400016 China

**Keywords:** Xpert MTB/RIF, Tuberculous pleurisy, Pleural fluid, Rifampicin resistance, Systematic review

## Abstract

**Background:**

Tuberculous pleurisy (TP) presents a diagnostic problem due to the limitations of traditional diagnostic methods. Different studies with the Xpert MTB/RIF assay have drawn variable conclusions about its values in TP diagnosis. We conducted a meta-analysis to assess whether the Xpert MTB/RIF assay is appropriate for the diagnosis of TP using pleural fluid samples.

**Methods:**

A systematic search of four literature databases in English and Chinese language was performed to identify studies involving the use of Xpert MTB/RIF in patients with TP confirmed by plural biopsy and/or mycobacterial culture. Pooled sensitivity, specificity and accordance proportion were calculated, and the forest plots were generated to assess the accuracy of Xpert MTB/RIF for TP diagnosis.

**Results:**

We identified 23 studies meeting our inclusion criteria. The pooled sensitivity and specificity of Xpert MTB/RIF were 30% (95% CI: 21–42%, I^2^ = 87.93%) and 99% (95% CI: 97–100%, I^2^ = 96.20%), respectively, and the area under the SROC curve (AUC) of Xpert MTB/RIF was 0.86 (95% CI: 0.83–0.89). Compared with drug susceptibility testing (DST), the pooled accordance rate of Xpert MTB/RIF in detecting rifampicin-susceptible cases and rifampicin-resistant cases was 99% (95% CI: 95–104%, I^2^ = 8.7%) and 94% (95% CI: 86–102%), respectively.

**Conclusions:**

Our analysis suggests that the Xpert MTB/RIF assay is of limited value as a screening test for TP but has a high potential for confirming TP diagnosis and differentiating TP from non-TB diseases using pleural fluid samples.

**Electronic supplementary material:**

The online version of this article (10.1186/s12879-018-3196-4) contains supplementary material, which is available to authorized users.

## Background

Tuberculosis (TB) remains a serious, life-threatening disease worldwide, with nearly 10.4 million cases and 1.7 million deaths reported in 2016 by the World Health Organization (WHO) [1]. While pulmonary TB is the most common presentation, extra-pulmonary TB is also an important clinical problem. One of the most common types of extra-pulmonary TB is tuberculous pleurisy (TP), which accounts for about one fourth of all TB cases [2]. At present, diagnosis of TP depends largely on detection of *Mycobacterium tuberculosis* in pleural fluid or pleura by microbiological culture, or demonstration of caseous granulomas in pleura by histopathological examination. However, these methods are invasive, laborious, time-consuming and insensitive, which often delay diagnosis and treatment.

Over the past several years, there has been a significant increase in using the Xpert MTB/RIF assay (also referred to as Xpert; Cepheid Inc., USA), which is an automated, cartridge-based nucleic acid amplification test for TB. This assay has the ability to simultaneously detect *M. tuberculosis* nucleic acid and resistance to rifampin (RIF) in less than 2 h. Due to its excellent performance, this assay has been recommended by WHO for diagnosing TB and detecting rifampicin resistance in pulmonary and extra-pulmonary TB in adults and children as well as for initial screening of individuals suspected of having multiple drug resistant-TB (MDR-TB) and HIV-co-infected TB cases [3, 4]. Most of the reported studies on Xpert MTB/RIF have been performed in sputum samples from pulmonary TB while there are relatively scarce reports using other types of samples from extra-pulmonary TB.

A limited number of studies have reported the utility of Xpert MTB/RIF in diagnosing TP, with highly variable sensitivity and specificity, ranging from 13 to 100%, between studies [5–7]. In one study carried out in pleural tissue samples from 17 patients with TP, Xpert MTB/RIF failed to detect any TP cases [8]. It is known that mycobacterial culture has limited ability to detect TP, when the study used only a culture reference standard and without histological biopsy, it is likely to overestimate the sensitivity of Xpert and underestimate the specificity [5, 9]. Although there is a published meta-analysis on the performance of Xpert MTB/RIF in diagnosing TP [10], this study has the limitations of using non-stringent inclusion criteria for TP patients (particularly without histopathological findings) and not including data on rifampicin-resistance. Therefore, the applicability of Xpert MTB/RIF to the diagnosis of TP as well as the detection of rifampicin-resistance in TP patients remains largely unclear.

To better understand the value of Xpert MTB/RIF in TP diagnosis, we conducted a comprehensive meta-analysis of literature published up to May 2018 involving the use of Xpert MTB/RIF for detecting TB and rifampicin resistance in TP patients.

## Methods

Our meta-analysis was presented with reference to the recommendations from the PRISMA statement [11]. All data involved in this analysis were extracted from published articles, therefore, ethical approval was not applicable in this study.

### Data sources and search strategy

A systematic search about studies of the accuracy of the Xpert MTB/RIF in diagnosing TP and rifampicin resistance was carried out. We searched the EMBASE, Cochrane, MEDLINE (PubMed) and China Science and Technology Journal (CSTJ) databases to identify original research articles and conference abstracts in English or Chinese language published on or before May 25, 2018. Search was implemented by using combinations of the following items: “pleural tuberculosis”, “tuberculous pleuritis”, “tuberculous pleural effusion”, “TPE”, “Xpert MTB/RIF”, “GeneXpert”, “Xpert”, and “TB/RIF”.

### Reference standard and study selection

Our literature search was restricted to studies involving the use of Xpert MTB/RIF in patients diagnosed as TP according to the current gold standard: a combination of histopathological examination and mycobacterial culture [2, 12]. That is: the reference standard of confirmed TP should include positive *Mycobacterium tuberculosis* culture from pleural fluid or tissue, or/ and histological manifestations of granulomas in pleural tissue.

As the first step, two investigators (ZY.Huo and L.Peng) independently screened for articles containing the defined items in the title and abstract. All articles reporting the performance of Xpert MTB/RIF in diagnosing TP and rifampicin resistance on pleural fluid samples were retrieved for full-text review. Through full-text review, articles were excluded if they met any of the following criteria: 1) Experiments were not performed with a commercial Xpert MTB/RIF assay; 2) The performance of Xpert MTB/RIF was not evaluated; 3) TP cases were not defined using a combination of histopathological examination and mycobacterial culture; 4) Xpert MTB/RIF was performed using blood or non-pleural fluid specimens; 5) Duplicated reports from the same research group; 6) Reports of systematic reviews or meta-analysis. After full-text review, the two researchers met together to compare the retrieved articles meeting the inclusion criteria. In case of any discrepancy, a third person (physician from our hospital) was invited to discuss and resolve the discrepancy.

### Data extraction and quality assessment

Two researchers (ZY.Huo and L.Peng) independently extracted and crosschecked the data from all included articles. The following information was retrieved from all included articles: 1) The first author, publication year and study location; 2) The number and age of the enrolled participants; 3) The proportion of HIV-seropositive participants; 4) The specimen types and diagnostic standard; 5) The statistics of positive and negative results of the Xpert MTB/RIF assay; 6) The statistics of rifampicin sensitive and resistant cases for the Xpert MTB/RIF assay. The quality of all included articles was evaluated based upon the recommendations from QUADAS-2 checklist [13]. Based on the QUADAS-2 system, concerns with respect to applicability and risk of bias in meta-analysis were verbalized as “high”, “unclear” or “high”. Two researchers (ZY.Huo and L.Peng) independently scored and recorded all included references using the QUADAS-2 tool, and then reviewed the results together. Next, IBM SPSS software 19.0 (SPSS Inc., USA) was used to calculate the kappa statistic for consistency check. In case of discrepancy, a third person (statistician from our university) was invited to re-evaluate the data and solve the disagreement.

### Statistical analysis

We performed descriptive statistics and analyses adopting the recommended methods for assessment of diagnostic trials in meta-analysis [14, 15]. All data analysis including the pooled sensitivity, specificity, and accordance proportion with corresponding 95% confidence intervals (95% CI), and the I^2^ statistic test was performed using the Stata/MP 13.1 (Stata Corp., USA). The results were summarized and synthesized by using forest plots. A symmetric receiver operator characteristic (SROC) curve was made to present the individual assessment of sensitivity and specificity for each study [16–18]. The publication bias of inclusive researches was evaluated by Deeks’ funnel plot asymmetry test.

## Results

### Literature search results and characteristics of published studies

**Fig. 1:**
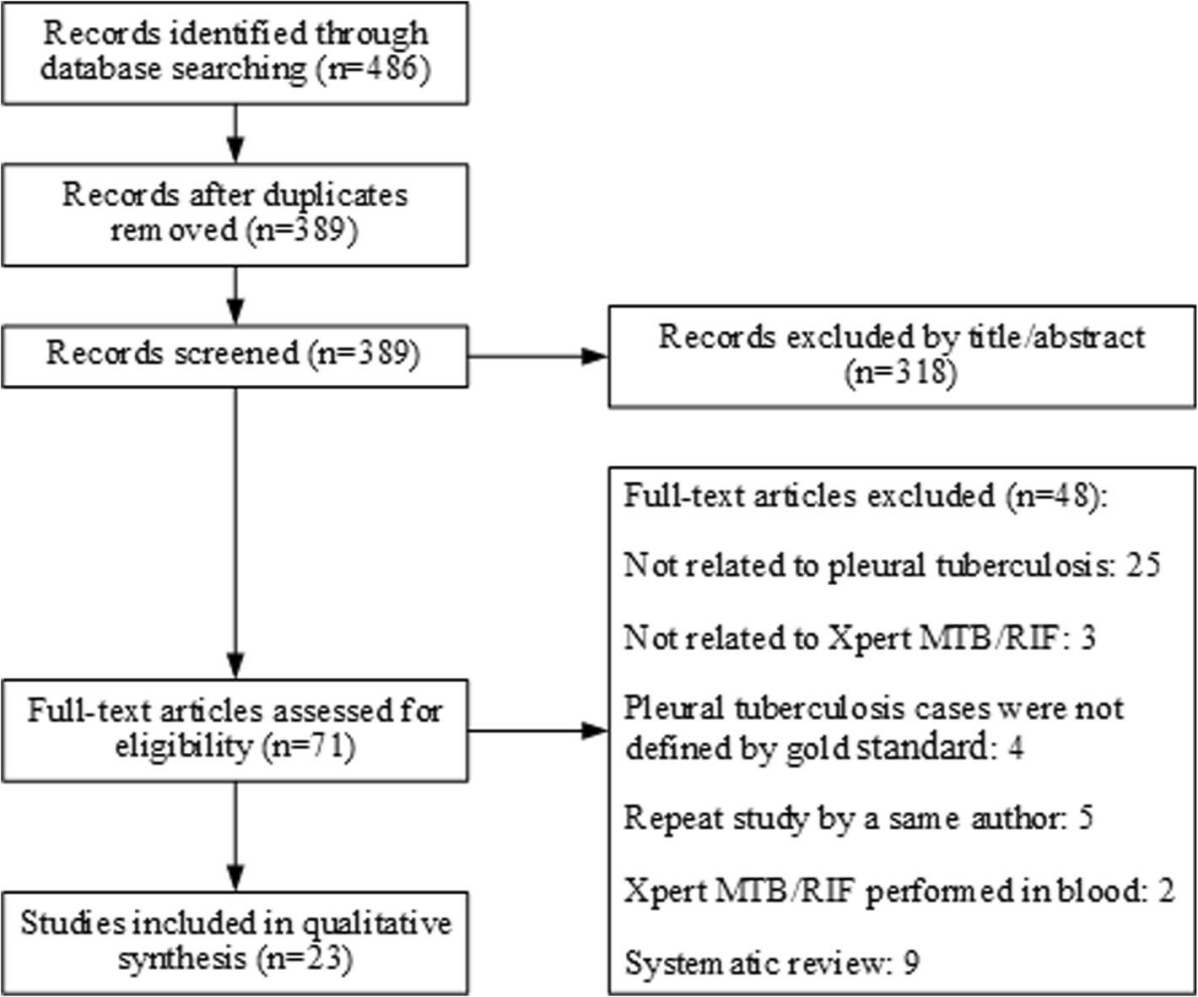
Flowchart diagram of the literature search process

Initial literature search resulted in a total of 486 unique studies. Following full-text review, we identified 23 studies that met all search criteria and were suitable for meta-analysis [5–9, 19–36]. Details of the literature selection process are illustrated in Fig. 1. The characteristics of the 23 included studies are summarized in Table 1. All these 23 studies demonstrated the performance of Xpert MTB/RIF in detecting TB or rifampicin resistance in TP patients confirmed following the gold standard involving a combination of mycobacterial culture and histopathological examination of pleural samples. Publication years ranged from 2011 to 2018 (Table 1). Study locations included 11 countries across Europe, South America, Asia and Africa. There was a total of 2646 individuals with pleural effusion, including 1194 (45.1%) with confirmed TP and 1452 (54.9%) without TP. Of the 23 studies, 7 reported the utility of Xpert MTB/RIF in HIV-associated TB patients, and 2 reported results of Xpert MTB/RIF in detection of rifampicin resistance in comparison with drug susceptibility testing (DST) [9, 31] (Table 2).

**Table 1:** Characteristics of 23 published studies included for meta-analysis and the primary results of Xpert MTB/RIF test

First author	Year	Country	Patients enrolled	Mean age (years)^a^	HIV-infection prevalence (%)	Specimen types	Reference standard	Results			
								TP	FP	FN	TN
Causse	2011	Spain	34	45(5–83)	NR	Pleural fluid	Culture	4	0	0	30
Friedrich	2011	South Africa	25	NR	NR	Pleural fluid	Culture	5	0	15	5
Malbruny	2011	France	12	52	4.3%	Pleural fluid	Culture	0	0	2	10
Moure	2012	Spain	31	NR	NR	Pleural fluid	Culture	7	0	19	5
Tortoli	2012	Italy	330	NR	NR	Pleural fluid	Culture	5	3	10	312
Christopher	2013	India	91	46(33–57)	NR	Pleural fluid	Culture and biopsy	4	0	26	61
Porcel	2013	Spain	67	50	0%	Pleural fluid	Culture and biopsy	5	0	28	34
Zmak	2013	Croatia	42	NR	NR	Pleural fluid	Culture	0	0	1	41
Javed	2014	Pakistan	25	NR	NR	Pleural fluid	Biopsy	2	0	12	11
Lusiba	2014	Uganda	116	34 ± 13	44.8%	Pleural fluid	Culture and biopsy	25	1	62	28
Meldau	2014	South Africa	88	51	10.2%	Pleural fluid	Culture and biopsy	9	1	31	47
Scott	2014	South Africa	528	39	NR	Pleural fluid	Culture	227	3	255	43
Sharma SK	2014	India	364	NR	NR	Pleural fluid	Culture	37	8	54	265
Theron	2014	South Africa	76	55(38–65)	17%	Pleural fluid	Culture	5	6	11	54
Trajman	2014	Brazil	59	50	5%	Pleural fluid	Culture and biopsy	1	0	32	26
Coleman	2015	Malawi	50	32	100%	Pleural fluid	Culture	9	0	4	18
Liu	2015	China	126	38.6 ± 13.2	4.0%	Pleural fluid	Culture	24	1	31	70
Rufai	2015	India	162	41.6 ± 19	0%	Pleural fluid	Culture	23	0	19	119
Wang	2015	China	125	43	NR	Pleural fluid	Culture	13	47	0	65
Che	2017	China	78	44 (18–83)	1.3%	Pleural fluid	Culture and biopsy	12	0	48	18
Li	2018	China	70	42 ± 20	0%	Pleural fluid	Culture and biopsy	6	0	39	25
Sharma S	2018	India	37	39	0%	Pleural fluid	Culture and biopsy	5	0	2	30
Christopher	2018	India	130	50.9 ± 14.1	0%	Pleural fluid	Biopsy	9	0	56	65

TP, true positive. FP, false positive. FN, false negative. TN, true negative. NR, not reported in the study

^a^ The values represent the means±SD or medians with corresponding interquartile ranges (IQRs)

**Table 2:** Characteristics and results of Xpert MTB/RIF studies on rifampicin resistance

First author	Year	Country	Samples enrolled	Xpert MTB/RIF		DST	
				Rifampicin sensitive	Rifampicin resistant	Rifampicin sensitive	Rifampicin resistant
Liu	2015	China	43	32	11	33	10
Wang	2015	China	13	13	0	13	0

DST, drug susceptibility testing

### Quality assessment of the included studies

Based on quality assessment by the QUADAS-2 tool, 11 of the 23 included studies showed a high risk of bias, whereas 5 of them had high applicability concerns. The kappa statistic between the primary results of two researchers (ZY.Huo and L.Peng) was 0.787 (P<0.01), which suggested a good inter-rater reliability of consistency. Additional information about the ratings of risk of bias and applicability concerns was provided in Additional file 1.

### Sensitivity and specificity of Xpert MTB/RIF

**Fig. 2:**
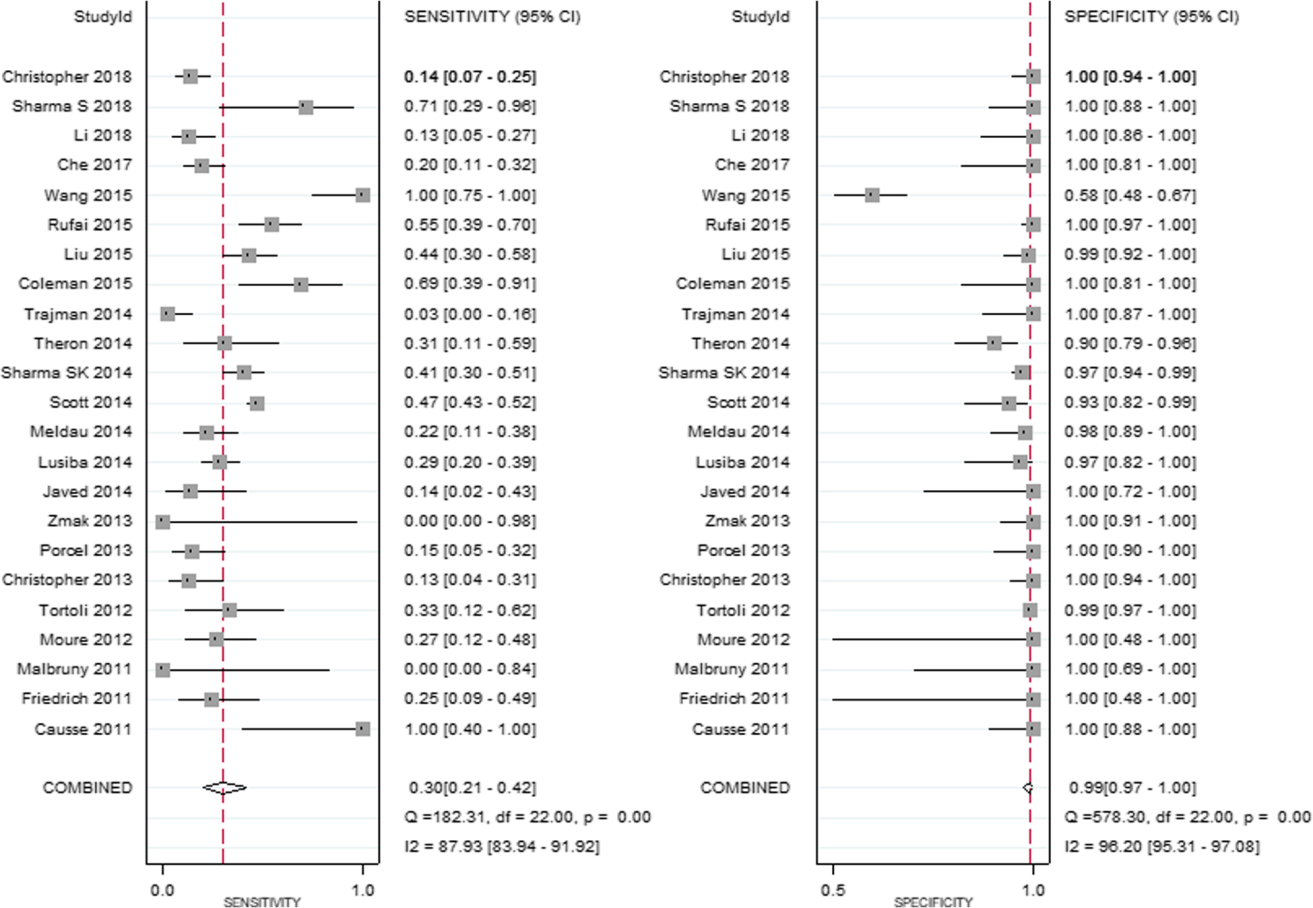
Forest plots of the performance of Xpert MTB/RIF in diagnosing TP. See references [5–9, 19–36] for details

**Fig. 3:**
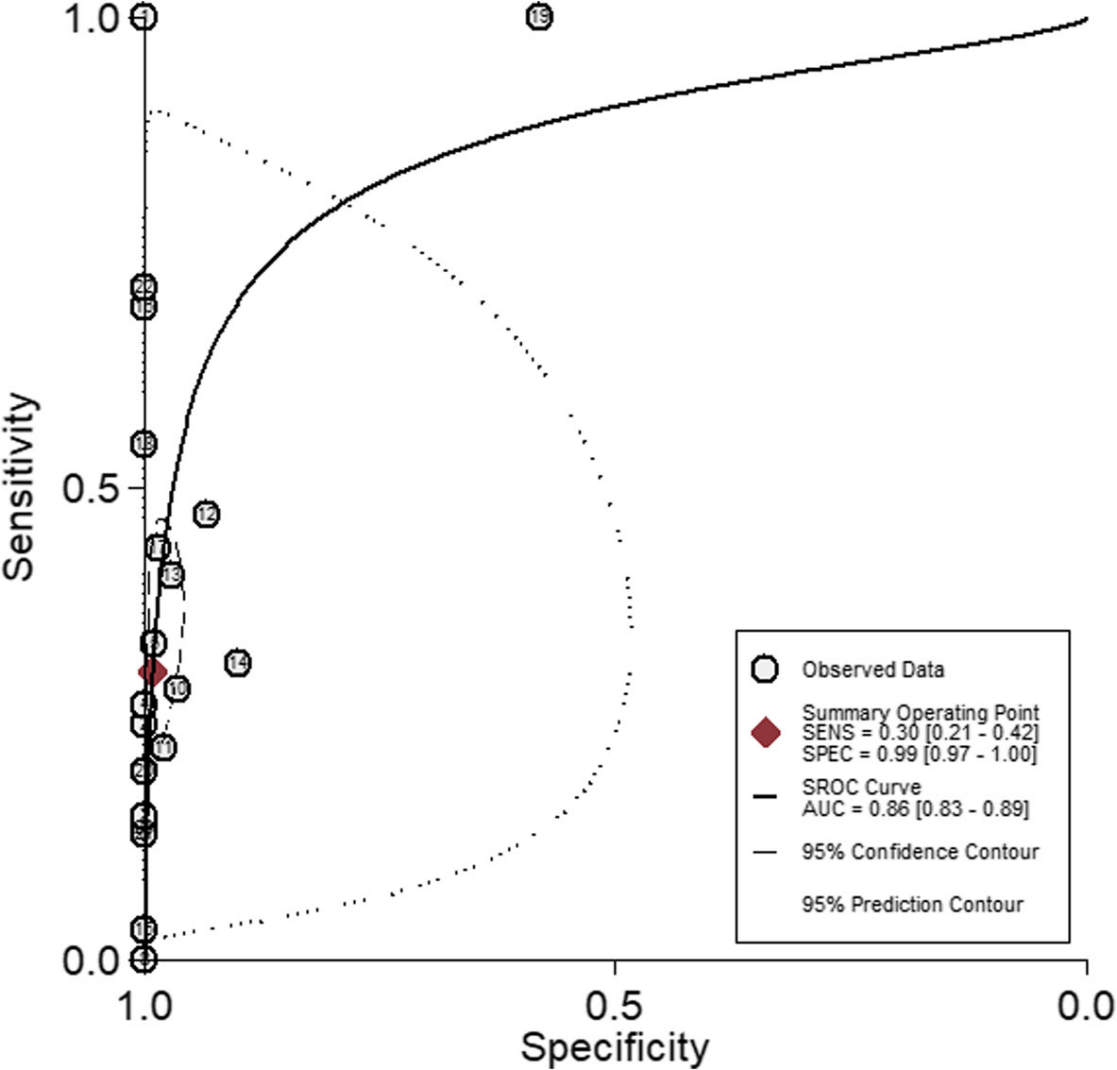
Symmetric receiver operator characteristic (SROC) curve for Xpert MTB/RIF assay. The SROC curve was derived by Stata/MP 13.1

A total of 2646 eligible participants were used to evaluate the performance of Xpert MTB/RIF for TP diagnosis, including 1194 participants confirmed to be TP by mycobacterial culture and/or histopathological examination, and 1452 participants not diagnosed as TP by the same criteria. For all these participants, pleural fluid samples were used in the Xpert MTB/RIF assay. The pooled sensitivity of Xpert MTB/RIF was 30% (95% CI: 21–42%, I^2^ = 87.93%), while the pooled specificity was 99% (95% CI: 97–100%, I^2^ = 96.20%, Fig. 2). The SROC curve for Xpert MTB/RIF was situated near the desirable upper left corner of the plot (Fig. 3), and the area under the SROC curve (AUC) was 0.86 (95% CI: 0.83–0.89).

### Accordance rate of rifampicin-susceptible and rifampicin-resistant cases

**Fig. 4:**
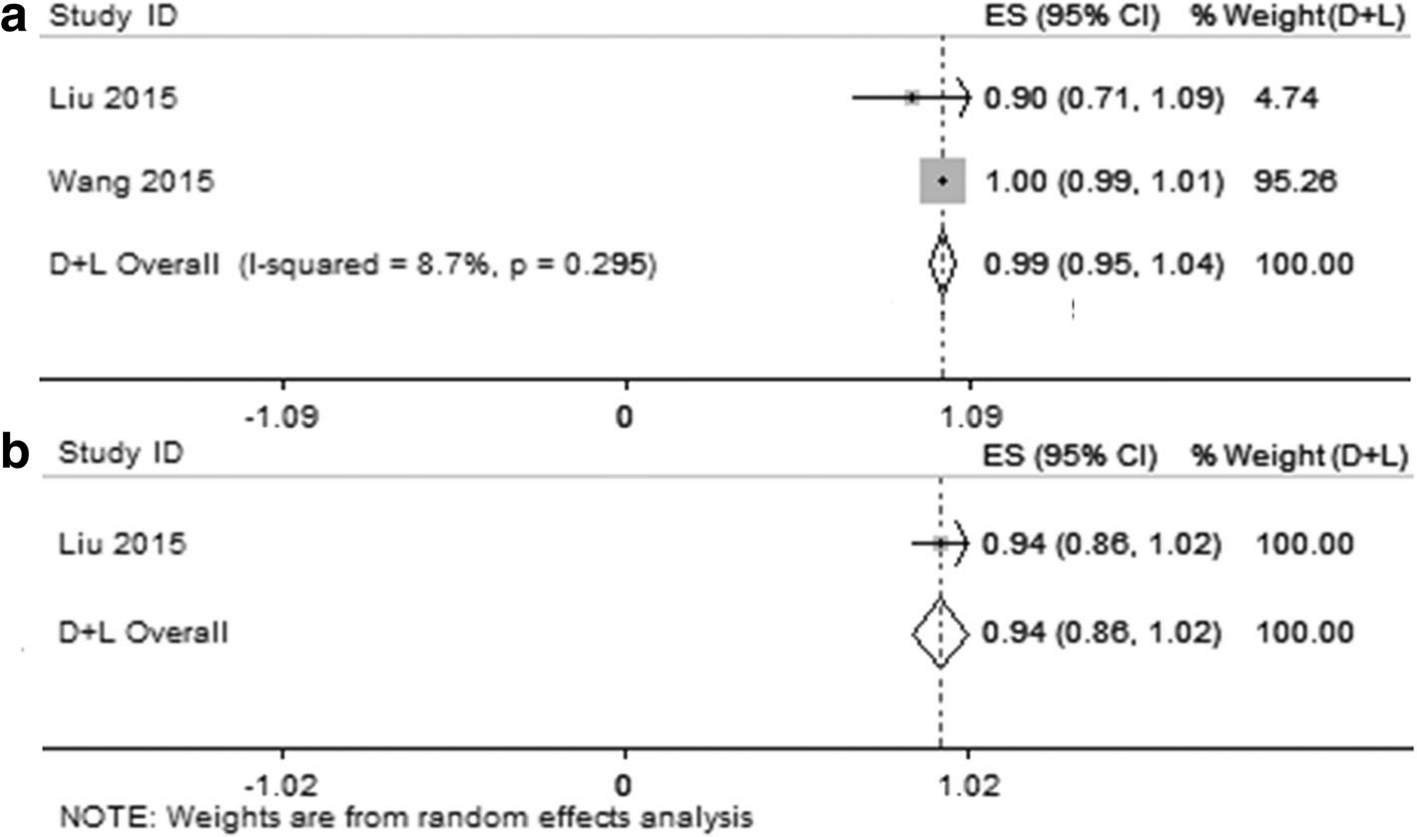
Forest plots of the pooled accordance rate of the Xpert MTB/RIF and DST test results for rifampicin-susceptible cases (**a**) and rifampicin-resistant cases (**b**). See references [9, 31] for details

Two of the 23 included studies presented the performance of Xpert MTB/RIF for the detection of rifampicin susceptibility and resistance in TP patients [9, 31]. Comparison of the results between Xpert MTB/RIF and DST revealed a pooled accordance rate of was 99% (95% CI, 95–104%, I^2^ = 8.7%) in rifampicin-susceptible cases, and 94% (95% CI, 86–102%) in rifampicin-resistant cases (Fig. 4). In one of these two studies [31], there was no rifampicin resistant case for either Xpert MTB/RIF or DST (Table 2), and thus the accordance rate was not calculated (Fig. 4b). These results indicate a trend of high concordance of Xpert MTB/RIF with DST for detection of rifampicin susceptibility and resistance, although the limited number of inclusive studies.

### Publication bias

**Fig. 5:**
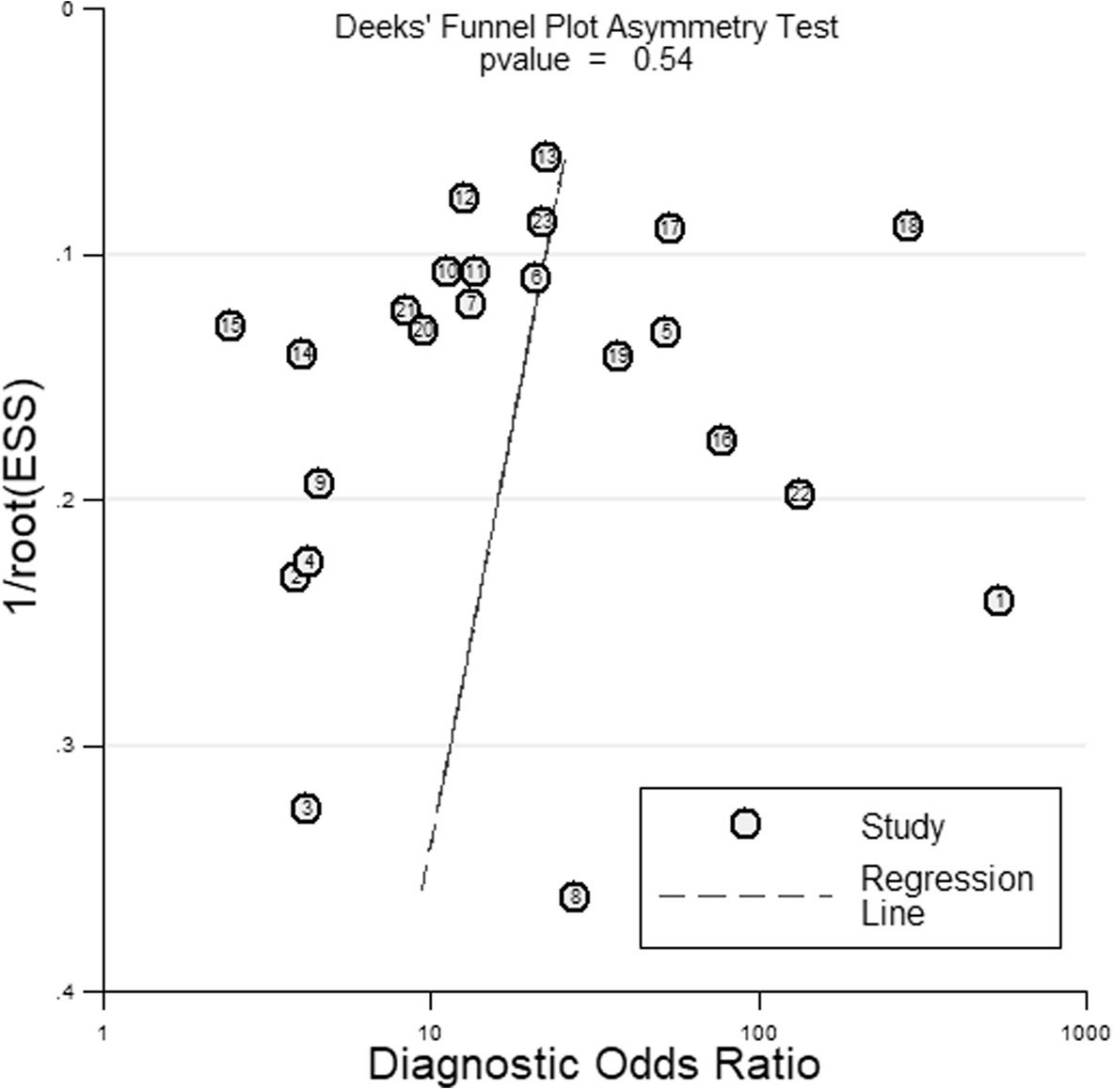
A Deeks’ funnel plot asymmetry test for evaluation of potential publication bias in Xpert MTB/RIF studies. This plot indicated a low risk of publication bias

Based on Deeks’ funnel plot asymmetry test, there was no significant asymmetry for the 23 studies on Xpert MTB/RIF (*P* = 0.54) included in our meta-analysis, suggesting a low risk for publication bias (Fig. 5).

## Discussion

Despite an increasing and widespread use of Xpert MTB/RIF for the diagnosis of pulmonary and extra-pulmonary TB worldwide, it remains unclear how valuable this method is for diagnosing TB and detecting rifampicin resistance in TP patients using pleural fluid samples. To summarize the results of and to overcome the limitations of small samples size among existing individual studies, we conducted this systemic review and meta-analysis to assess the performance of Xpert MTB/RIF in diagnosing TP and detecting rifampicin susceptibility or resistance using pleural fluid specimens. In order to produce the best possible results, we adopted a combination of histopathological examination and mycobacterial culture as the reference standard, which is the current gold standard for the diagnosis of TP. Our meta-analysis involves a total of 2646 patients with pleural effusion from 23 eligible studies published between 2011 and 2018 among 11 countries across Europe, South America, Asia and Africa (Table 1).

Based on our meta-analysis, the overall sensitivity and specificity of Xpert MTB/RIF in diagnosing TP from 23 eligible studies were 30 and 99%, respectively, indicating a low sensitivity and a high specificity. While the high specificity of this assay suggests it to be an excellent rule-in test (confirming TP diagnosis), the low sensitivity suggests a limited rule-out value (ruling out TP diagnosis). The ability of Xpert MTB/RIF to detect rifampicin susceptibility or resistance was evaluated in two small studies, which showed a trend of high concordance with DST, suggesting its potential usefulness for detection of MDR-TB in TP patients.

A similar meta-analysis has been previously reported by Sehgal et al. [10], which reported a pooled sensitivity and specificity of 51.4 and 98.6%, respectively, with culture used as a reference standard, and 22.7 and 99.8%, respectively, with a composite reference standard (CRS) used as the benchmark. The specificity in this report is similar to that in our analysis (99%) while the sensitivity in this report appears to be different compared to our analysis (30%). The exact reasons for the difference in sensitivity are unclear. One possible explanation is the lack of histopathological examination in the reference standard in the report of Sehgal et al. [10], which may lead to different results in sensitivity compared to our analysis. We used a combination of mycobacterial culture and histopathological examination as the reference standard, and excluded 9 studies included in the report of Sehgal et al. [10], which did not meet our reference standard while adding 8 new studies [9, 23, 30, 31, 33–36] in our analysis. Given that the reference standard we used is the current gold standard, it is likely that the results of our analysis more reliable.

Nevertheless, among the 23 studies included in our analysis there was substantial heterogeneity (I^2^ = 87.93 and 96.20% for sensitivity and specificity, respectively). One prominent example is the study by Wang et al. [9], which showed an exceptionally high sensitivity but a very low specificity as clearly shown in the forest plots in Fig. 2. The reason for this observation is uncertain but could be related to the use of mycobacterial culture alone as the reference standard in this study. Since the mycobacterial culture method is known to have low sensitivity in TB diagnosis, evaluation of Xpert MTB/RIF using samples from patients confirmed by culture alone is likely to overestimate the sensitivity and underestimate the specificity, as has been reported previously [5, 9].

Although there is apparent variation in the sensitivity of Xpert MTB/RIF among different studies on TP patients, the overwhelming trend is a low sensitivity (around 30%). When this assay is used as a diagnostic test, approximately 70% of TP patients could be misdiagnosed, suggesting that this assay is not appropriate as an initial screening test for suspected TP patients in countries with high TB burden. The reasons for this low sensitivity remain poorly understood, but could be due to the presence of PCR inhibitors in pleural fluid samples, and use of inappropriate or inefficient sampling methods [37]. Clearly, further research is needed to optimize the sample processing in order to improve the sensitivity of Xpert MTB/RIF.

Despite a low sensitivity for diagnosing TP, Xpert MTB/RIF consistently showed an excellent specificity (99%), which is attributed largely to the use of highly specific genetic target in this test. The high specificity suggests its high value in confirming TP diagnosis and differentiating TP from non-TB diseases, especially in countries with low or intermediate TB burden.

For the Xpert MTB/RIF assay in detection of rifampicin resistance in pleural fluid samples, the pooled accordance rate of rifampicin-susceptible cases and rifampicin-resistant cases was 99 and 94%, separately. The high concordance between the Xpert MTB/RIF and DST indicated good efficiency for rifampicin resistance detection, which was similar to the previous studies [38–40]. Although the number of inclusive studies in our meta-analysis is limited, the result indicated the Xpert MTB/RIF could rapidly detect patients suffered from MDR-TB and give them rapid initiation of anti-MDR-TB therapy.

The Deeks’ funnel plot asymmetry test was performed to evaluate and analyze the publication bias among 23 inclusive studies (Fig. 5). The plot showed no significant asymmetry for the Xpert MTB/RIF (*P* = 0.54), and there was no evidence of potential risk of publication bias.

## Conclusions

In summary, the results of our meta-analysis suggest that the Xpert MTB/RIF assay is of limited value as a screening test for TP but has a high potential for confirming TP diagnosis and differentiating TP from non-TB diseases. The Xpert MTB/RIF assay showed high concordance with DST, suggesting its usefulness for detection of MDR-TB, which may help early decision making in anti-MDR-TB therapy.

## Additional file

Additional file 1: Methodological quality evaluation results of 23 studies sorted using the Quality Assessment of Diagnostic Accuracy Studies-2 (QUADAS-2) tool. The ratings of risk of bias and applicability concerns were provided. (TIF 3222 kb)

## Abbreviations

95%CI: 95% confidence intervals
AUC: Area under the SROC curve
DST: Drug susceptibility testing
MDR-TB: Multiple drug resistant-TB
PCR: Polymerase chain reaction
PRISMA: Preferred reporting items for systematic reviews and meta-analyses
QUADAS-2: Quality Assessment of Diagnostic Accuracy Studies-2
RIF: Rifampin
SROC: Symmetric receiver operator characteristic
TB: Tuberculosis
TP: Tuberculous pleurisy
WHO: World Health Organization
